# Computational Analysis of Plasma Lipidomics from Mice Fed Standard Chow and Ketogenic Diet

**DOI:** 10.21769/BioProtoc.4819

**Published:** 2023-09-20

**Authors:** Amy L. Seufert, James W. Hickman, Jaewoo Choi, Brooke A. Napier

**Affiliations:** 1 Department of Biology and Center for Life in Extreme Environments, Portland State University, Portland, OR, USA; 2 Linus Pauling Institute, Oregon State University, Corvallis, OR, USA

**Keywords:** Lipidomics, Mass spectrometry, Ketogenic diet, Free fatty acids, Phosphatidylcholines, Triglycerides, Sphingolipids, Circulating lipids

## Abstract

Dietary saturated fatty acids (SFAs) are upregulated in the blood circulation following digestion. A variety of circulating lipid species have been implicated in metabolic and inflammatory diseases; however, due to the extreme variability in serum or plasma lipid concentrations found in human studies, established reference ranges are still lacking, in addition to lipid specificity and diagnostic biomarkers. Mass spectrometry is widely used for identification of lipid species in the plasma, and there are many differences in sample extraction methods within the literature. We used ultra-high performance liquid chromatography (UPLC) coupled to a high-resolution hybrid triple quadrupole-time-of-flight (QToF) mass spectrometry (MS) to compare relative peak abundance of specific lipid species within the following lipid classes: free fatty acids (FFAs), triglycerides (TAGs), phosphatidylcholines (PCs), and sphingolipids (SGs), in the plasma of mice fed a standard chow (SC; low in SFAs) or ketogenic diet (KD; high in SFAs) for two weeks. In this protocol, we used Principal Component Analysis (PCA) and R to visualize how individual mice clustered together according to their diet, and we found that KD-fed mice displayed unique blood profiles for many lipid species identified within each lipid class compared to SC-fed mice. We conclude that two weeks of KD feeding is sufficient to significantly alter circulating lipids, with PCs being the most altered lipid class, followed by SGs, TAGs, and FFAs, including palmitic acid (PA) and PA-saturated lipids. This protocol is needed to advance knowledge on the impact that SFA-enriched diets have on concentrations of specific lipids in the blood that are known to be associated with metabolic and inflammatory diseases.

Key features

• Analysis of relative plasma lipid concentrations from mice on different diets using R.

• Lipidomics data collected via ultra-high performance liquid chromatography (UPLC) coupled to a high-resolution hybrid triple quadrupole-time-of-flight (QToF) mass spectrometry (MS).

• Allows for a comprehensive comparison of diet-dependent plasma lipid profiles, including a variety of specific lipid species within several different lipid classes.

• Accumulation of certain free fatty acids, phosphatidylcholines, triglycerides, and sphingolipids are associated with metabolic and inflammatory diseases, and plasma concentrations may be clinically useful.

**Graphical overview**


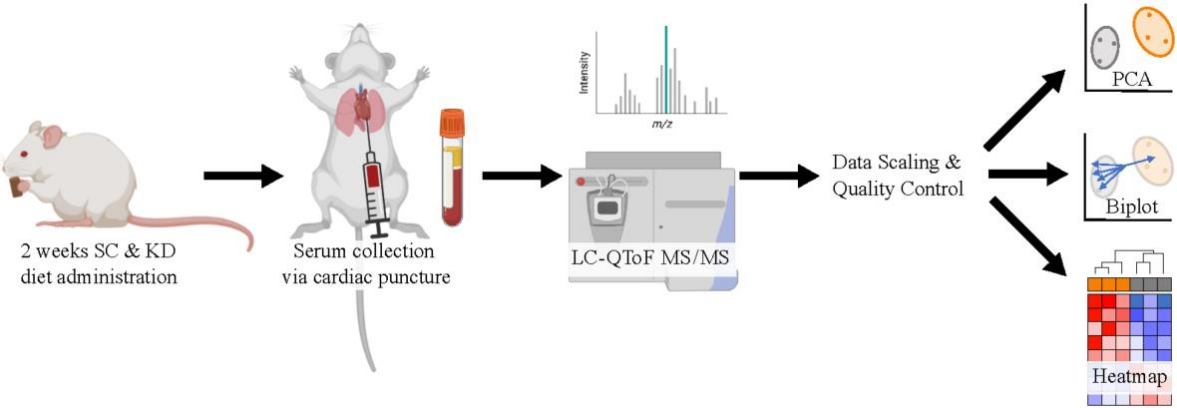


## Background

Circulating lipids in the blood, specifically cholesterol and triglycerides (TAGs), are measured in the clinic to determine cardiovascular disease (CVD) risk (Sarwar et al., 2007; Ference et al., 2017). Additional classes of lipids, including free fatty acids (FFAs), phosphatidylcholines (PCs), and sphingolipids (SGs), are associated with metabolic, inflammatory, and infectious diseases; however, reference ranges have not yet been determined. Within lipid classes, specific lipid species may also be useful as biomarkers for early detection of CVD, COVID-19, sepsis, cancer, neurodegenerative diseases, and chronic obstructive pulmonary disease (Kubicek-Sutherland et al., 2017; Kohno et al., 2018; Liu et al., 2020; Casas-Fernández et al., 2022; Ciccarelli et al., 2022). High-throughput lipidomics is a valuable tool for identifying a diversity of lipid species and their relative concentrations within a small plasma sample (< 50 μL). Here, we describe our protocol for analyzing a large dataset, collected via ultra-high performance liquid chromatography (UPLC) coupled to a high-resolution hybrid triple quadrupole-time-of-flight (QToF) mass spectrometry (MS), encompassing 15 different lipid classes, with a focus on four clinically relevant lipid species: FFAs, TAGs, PCs, and SGs (Choi et al., 2015; Seufert et al., 2022). The plasma samples measured were harvested from mice fed a standard chow (SC) or a ketogenic diet (KD) for two weeks. Using this protocol, we were able to determine relative abundances of FFAs, TAGs, PCs, and SGs, and take a snapshot of diet-induced lipidomic profiles within mice. This protocol may be used to analyze any omics dataset.

Currently, common blood lipid panels used in the clinic lack detail regarding specific lipid species found in the blood, measuring only low-density, very-low-density, and high-density lipoprotein cholesterol and total TAGs in order to identify hyperlipidemia, CVD risk and metabolic syndrome (Lee et al., 2023). Although plasma cholesterol and total TAG concentrations are useful in the prevention and identification of hyperlipidemia, CVD, and metabolic syndrome, many other circulating lipids exist that are known to correlate with inflammatory and metabolic dysfunction. For example, dietary saturated fats such as palmitic acid (PA) have recently been shown to sensitize innate immune cells to microbial ligand exposure in vitro and in vivo, and serum PA concentrations positively correlate with insulin resistance in humans (Perreault et al., 2014; Seufert et al., 2022). The methods outlined in this protocol and our corresponding publication (Seufert et al., 2022) show the efficacy of using high-throughput lipidomics to compare relative concentrations of specific lipid species in the blood of SC- and KD-fed mice. Our results highlight the importance of this protocol for future studies in mice or humans that aim to identify lipid changes in the blood due to SFA-enriched diets and supports the advancement of personalized nutrition for humans suffering from metabolic and inflammatory diseases.

**Figure 1.**
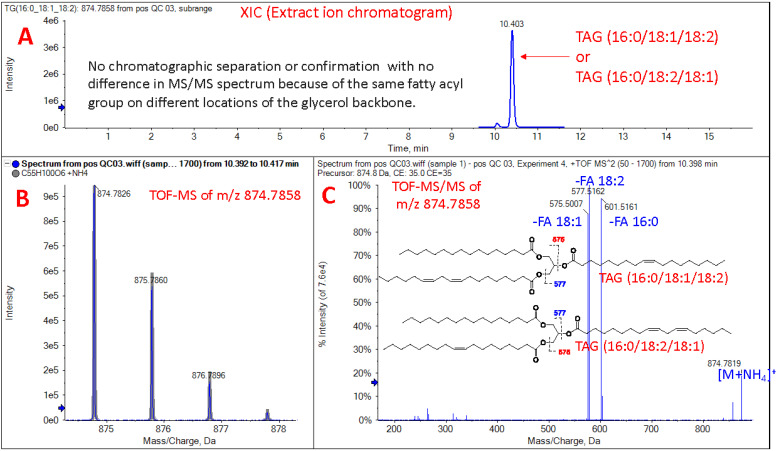
Isobaric structure of triglyceride as shown by UPLC-QToF MS/MS: TAG (16:0/18:1/18:2) or TAG (16:0/18:2/18:1). Separation and identification of isobaric lipids with identical molecular formulas is limited due to structural differences that do not show chromatographic separation. (A) Extract ion chromatogram of TAG (16:0/18:1/18:1) or TAG (16:0/18:2/18:1). (B) ToF-MS isotopic pattern of m/z 874.7858. (C) ToF-MS/MS of m/z 874.7858, which shows ammonium adduct in positive ion mode.

UPLC-QToF MS/MS identifies hundreds of lipid species within a single plasma sample; measuring the chromatographic peak area under the curve (AUC) for each lipid allows for relative comparisons within and between samples (Choi et al., 2015). Absolute concentrations may be determined for some lipids; however, specific MS standards at additional costs are required, and absolute concentrations were not measured in this study. Due to the numerous combinations of different headgroups and fatty acids including chain length, number, and position of double bonds, the separation and identification of isobaric lipids that have identical molecular formulas but structural differences are limited (Batarseh et al., 2018). For instance, TAG (16:0/18:1/20:4) and TAG (18:1/18:2/18:2) are isobaric lipids. These lipids are chromatographically separated and identified by MS/MS fragmentation. However, TAG (16:0/18:1/18:2) and TAG (16:0/18:2/18:1) are not chromatographically separated with no difference in MS/MS spectrum because of the same fatty acyl group on different locations of the glycerol backbone (Figure 1). The ion mobility or ozone-induced dissociation techniques can afford information on double bond and location of fatty acyl group on lipids. Lastly, aside from essential FAs that are known to be only derived from exogenous sources (omega-3 and omega-6), lipidomics data does not indicate whether plasma lipids entered the bloodstream directly following digestion of lipids or were produced endogenously by the host. Thus, proper controls are required when studying diet-dependent effects.

## Materials and reagents

**Biological materials**

1. Age-matched (4–6 weeks) female wildtype BALB/c mice; JAX stock #000651 (see General note 1)

**Reagents**

1. Heparin sodium (VWR, catalog number: AAA16198-03)
2. 1× PBS (Thermo Fisher, catalog number: 20012050)

**Recipes**

1. Heparin sodium, diluted to 100 IU/mL in 1× PBS, ~5 mL

**Laboratory supplies**

1. PicoLab Mouse Diet 20 (Irradiated chow; product 5058), softened with water for first week of acclimation
2. Compressed CO_2_ gas in cylinders for euthanizing mice (fill rate: 30%–70% of chamber vol/min)
3. Standard chow mouse diet (Envigo, TD.08485) (see General note 1)
4. Ketogenic mouse diet (Envigo, TD.180423) (see General note 1)
5. BD Vacutainer blood collection tubes (BD, catalog number: 366667)
6. 3 mL BD Luer-Lok syringe with attached needle (25 G × 5/8 in.) (BD, catalog number: 309570)
7. Axygen Maxy Clear Snaplock microcentrifuge tubes (VWR, catalog number: 10011-700)
8. Parafilm M wrapping film (Fisher Scientific, catalog number: S37440)
9. Ice and ice bucket

## Equipment

1. Tabletop centrifuge for microcentrifuge tubes set at 4 °C
2. Computer with a Microsoft OS (version dependent on PeakView and MultiQuant version utilized)
3. Euthanasia chamber for mice

## Software and datasets

1. PeakView version 1.2.1 (Sciex) (see General note 2)
2. MultiQuant version 3.0.2 (Sciex) (see General note 2)
3. Excel (Microsoft Office 2019) (see General note 2)
4. R Statistical Software (https://www.r-project.org)
5. RStudio open-source edition, Boston, MA, USA (https://www.rstudio.com)
6. GraphPad Prism 9 (www.graphpad.com)

## Procedure

1. Allow mice to acclimate undisturbed to research facility with soft food for one week prior to changing diets.
2. Separate mice and change diets to SC or KD two weeks prior to plasma collection date.
*Note: The KD is soft and requires refrigeration and daily food changes; cages must be cleaned or replaced every three days.*
3. Immediately prior to euthanizing mice, prepare heparin solution (~5 mL) and coat syringes for cardiac punctures by aspirating and expelling the solution with each syringe. The same heparin solution can be used for multiple syringes. After coating, syringes may be placed on a sterile surface resting on needle caps. Ensure that the needles are bevel up and ready for use.
4. Prepare blood collection tubes and ice bucket and set tabletop centrifuge temperature to 4 °C.
5. Euthanize mice one at a time with CO_2_ and prior to cervical dislocation, place mouse supine on bench, and quickly perform cardiac puncture using 3 mL BD Luer-Lok syringe with attached 25 G needle.
  It is important that the needle gauge used for this procedure is between 23 and 25 G, in order to avoid hemolysis. Hemolysis is the destruction of red blood cells, and it has been shown to significantly impact levels of certain lipid species in the blood (Burla et al., 2018).
6. Transfer blood (200–700 μL depending on size of mouse) to BD Vacutainer blood collection tubes and keep on ice.
7. Transfer blood samples to microcentrifuge tubes and centrifuge at 1,500× *g* for 20 min at 4 °C.
8. Collect transparent plasma (supernatant) and transfer to a fresh microcentrifuge tube.
  Seal tubes with parafilm and store at -80 °C or ship on dry ice for sample processing to: Jan F. Stevens & Jaewoo Choi. Linus Pauling Institute, Oregon State University, Corvallis, OR, USA (Choi et al., 2015). Frozen plasma (20 μL) was extracted with lipidomics extraction solvent (480 μL, methylene chloride:methanol:isopropanol = 25:10:65, v/v/v + 0.1% BHT), vortexed for 30 s, centrifuged for 10 min at 13,000 rpm at 4 °C. The aliquot (95 μL) was transferred into mass spectrometry analysis tubes and SPLASH LipidoMix (Avanti Lipids) as internal standard mixture (5 μL) was spiked. UPLC was performed using a 1.7 μm particle, 2.1 × 100 mm, CSH C18 Column (Waters, Milford, MA, United States) coupled to a quadrupole TOF mass spectrometer (AB SCIEX, TripleTOF 5600) operated in information-dependent MS/MS acquisition mode. LC and MS conditions were developed as described previously by Choi et al. (2015) with some modifications. For positive ion mode LC-QToF-MS/MS, the mobile phases consisted of (A) 60:40 (v/v) acetonitrile: water with ammonium formate (10 mM) and formic acid (0.1%) and (B) 90:10 (v/v) isopropanol:acetonitrile with ammonium formate (10 mM) and formic acid (0.1% formic acid). For analyses run in the negative ion mode, ammonium acetate (10 mM) was used as the modifier.

## Data analysis

**Identification and quantification of lipidomics**

**Figure 2.**
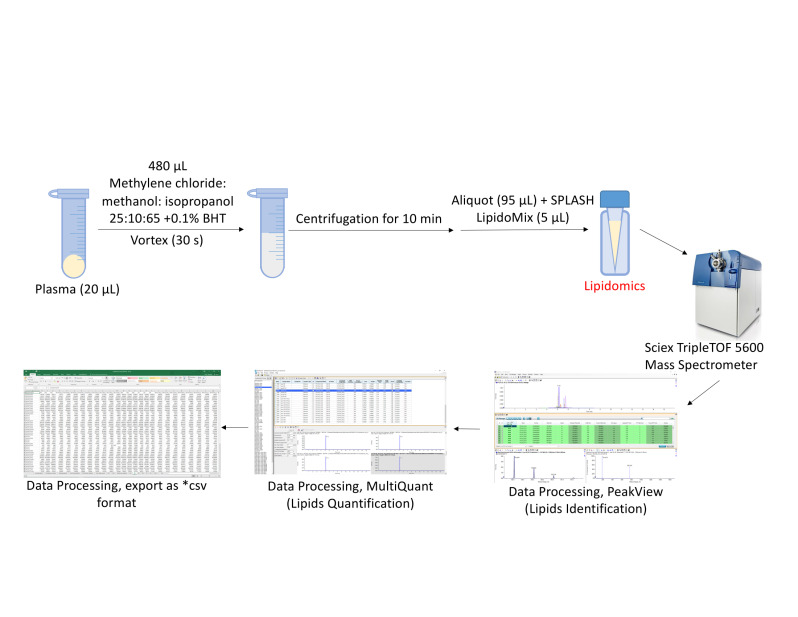
Lipidomics data processing

**Figure 3.**
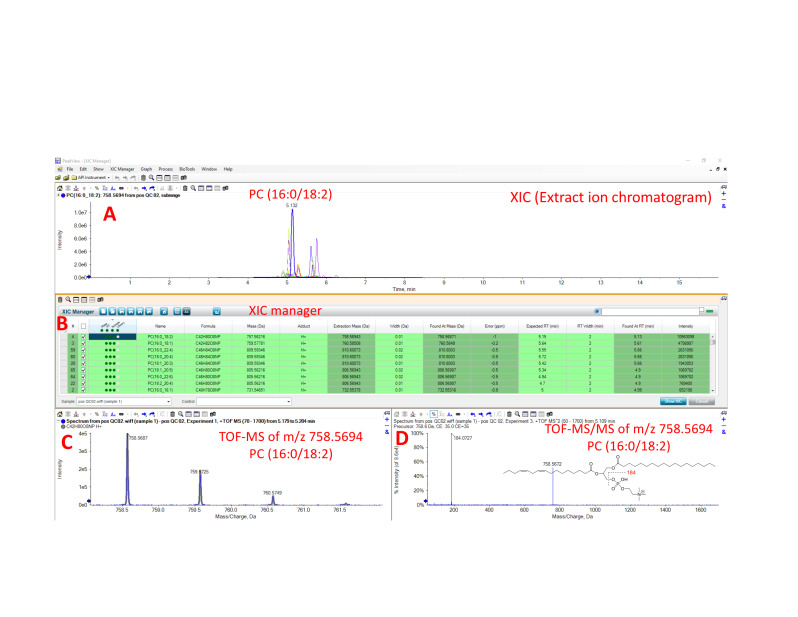
Lipid [PC (16:0/18:2)] identification using PeakView software, which explores and interprets qualitative data. (A) Extract ion chromatogram (XIC) of PC (16:0/18:2). (B) XIC manager displays in the table including found mass, mass error, found retention time, formula, adduct, and exact mass. (C) TOF-MS isotopic pattern of m/z 758.5694, which shows PC (16:2/18:0). (D) TOF-MS/MS of m/z 758.5694 as protonated adduct. The m/z 184 represents a unique fragment ion (protonated phosphocholine) corresponding to phosphatidylcholine.

**Figure 4.**
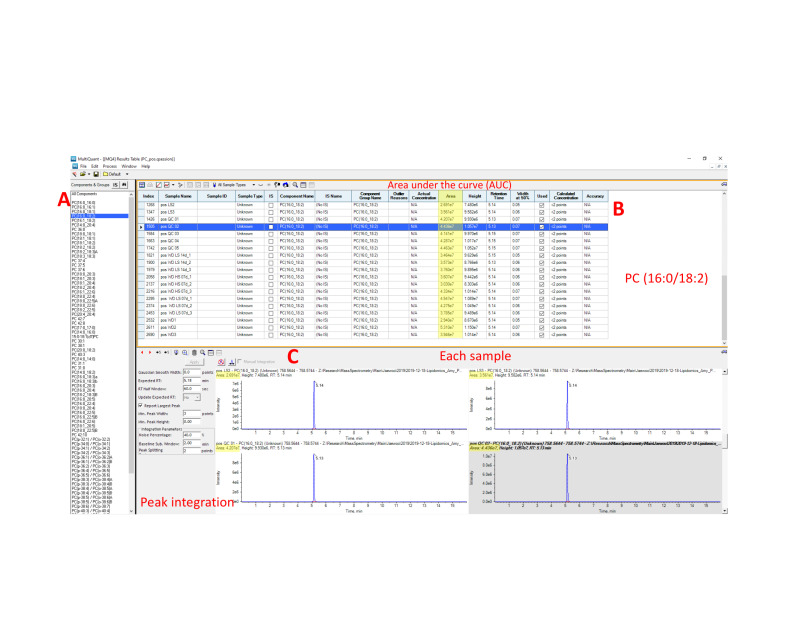
Chromatographic peak area integration using MultiQuant software. (A) Analyte pane includes identified PCs list. (B) Result pane includes each sample name and peak area counts with retention time. (C) Chromatogram review in each sample. The peak can be automatically or manually integrated. AUC values are highlighted in yellow.

1. Process the lipidomics data with PeakView 1.2.1 and quantify lipids with MultiQuant software version 3.0.2 (see General note 3, Choi et al., 2015). Raw MS files (*.wiff) were imported and processed by the program PeakView (Sciex) (Figure 2, Figure 3). PeakView detects spectral features using extract ion chromatogram (XIC) lists from our in-house library of lipids (each defined by a unique chromatographic retention time and accurate mass, MS/MS fragmentation, and isotopic pattern; Figure 3, B and C). Each peak was integrated by MultiQuant (Sciex) software (Figure 4). Peak integration is the quantification step whereby the peak area of an identified lipid is calculated. Peak area is referred to in this protocol as the AUC and this value is proportional to the quantity of the identified lipid.
2. Save lipidomics data as Excel or .csv files. Normalization can be done in Excel with the following formula: peak area ratio = peak area of identified lipid/peak area of labeled internal standard. Each integrated chromatogram was normalized manually with the use of an internal standard peak purchased from Avanti lipids (SPLASH LipidoMix) (Figure 4).

**Bioinformatic analysis in R**

1. In R, scale each lipid type or class dataset with the scale() function. For mean centering, replace NA values with the mean of the particular variable. For more detailed information on the code see General note 4.
2. Perform a PCA analysis with the prcomp() function.
  PCA analysis is a technique for multivariable data that performs dimension reduction to represent the most important and impactful information as principal components. This allows the data to be visually observed for patterns of similarity (Abdi and Williams, 2010).
3. Visualize the first two principal components for the dataset for each lipid type with the fviz_pca_ind() function from the factoextra package (Kassambara and Mundt, 2017). Color samples by diet group and create a confidence ellipse around each sample group (Figure 5A).
  Set addEllipses = TRUE, ellipse.type = “confidence,” and ellipse.level = 0.95 to create confidence ellipses.
4. Investigate strong separation between groups in the 2D space indicated by no overlap between the concentration ellipses. Sample groups with low separation for lipid types will have overlapping ellipses and data points.
5. Investigate specific lipid variables contributing to separation with fviz_pca_biplot() to create a biplot (Wickham, 2016) (Figure 5B).
  - Choose the top five contributing variables to view with select.var = list(contrib = 5). Adjust the number to increase or decrease the desired number of variables.
  - Phosphatidylcholine (PC) variables are lipids identified within each plasma sample represented by vectors that are close together and that form small angles with one another because they are positively correlated. Variables point in the direction of the principal component(s) they strongly influence. In the PC biplot, all of the variables point along the PC1 axis towards the KD samples, indicating that the KD samples are relatively more enriched in these PCs. This enrichment strongly contributes to the separation of the KD and SC, as the SC samples group towards the opposite side of PC1, away from where the variables are pointing.
6. Perform a heatmap analysis with hierarchical clustering using the pheatmap() function on the dataset (Kolde, 2018). Identify lipid types with clear clustering between diet groups (Figure 5C).
  **Figure 5.**
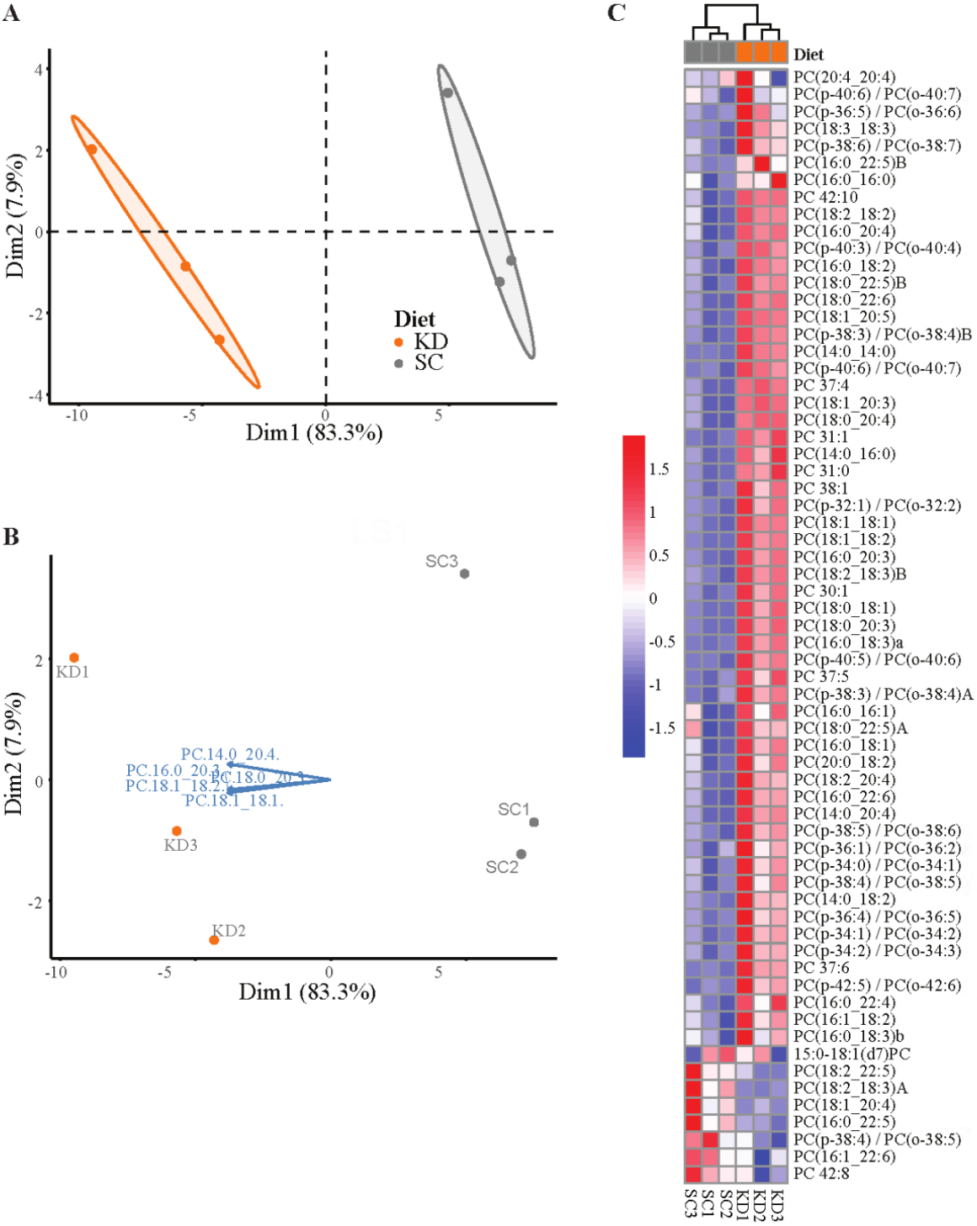
Lipidomics data analysis (see General note 4). (A) PCA plot of phosphatidylcholine composition identified via LC-QToF MS/MS between age-matched (6–8 weeks) BALB/c female mice. Dots represent individual mice with colors corresponding to a standard chow (SC) (grey) or ketogenic diet (KD) (orange) with the mean of each diet group surrounded by a 95% confidence ellipse. (B) Biplot labeled with the top five phosphatidylcholines contributing to sample separation in 2D space. (C) Heatmap analysis of phosphatidylcholine composition between individual mice.
7. Perform *t*-tests in R or, alternatively, GraphPad Prism to compare significance of lipids between sample diet groups (Table 1).
  **Table 1.** Phosphatidylcholine (PC) data and significance for standard chow (SC)- and ketogenic diet (KD)-fed mice shown in Figure 5. Statistical significance determined by unpaired two-tailed *t*-test between SC and KD groups, n = 3, AUC = area under the curve.

Phosphatidylcholine (PC)	SC (mean AUC)	KD (mean AUC)	p-value
PC 37:4	107,470	317,878	0.000183788
PC 31:1	2,019	11,590	0.000298222
PC(18:1_20:5)	350,900	1,037,459	0.000400427
PC(18:0_22:6)	1,532,375	5,075,115	0.000436107
PC(14:0_14:0)	853	13,466	0.000506701
PC(18:1_18:1)	9,425,085	46,602,806	0.000613854
PC(p-40:6)/PC(o-40:7)	35,344	72,388	0.000634691
PC(p-38:3)/PC(o-38:4)B	56,172	137,571	0.000665094
PC(18:1_20:3)	8,158,408	25,437,034	0.000693246
PC(18:0_20:4)	11,279,355	33,755,056	0.000713162
PC(18:1_18:2)	2,729,249	10,297,513	0.000744278
PC(16:0_20:3)	3,719,482	14,331,321	0.000806004
PC(p-40:3)/PC(o-40:4)	21,627	43,188	0.000929417
PC(18:0_20:3)	568,891	3,223,095	0.000986705
PC(16:0_18:3)	134,495	426,137	0.001061905
PC(18:2_18:3)B	203,100	527,797	0.001373022
PC(18:0_18:1)	915,812	6,979,001	0.001377977
PC(p-40:5)/PC(o-40:6)	52,554	141,136	0.001658554
PC 42:10	32,839	62,822	0.001987255
PC 30:1	2,889	15,436	0.00199227
PC 31:0	41,226	218,739	0.002324314
PC(16:0_18:2)	30,188,177	53,002,677	0.002454378
PC(18:0_22:5)	78,146	250,710	0.002602027
PC(14:0_16:0)	120,007	663,490	0.003213188
PC(14:0_20:4)	13,280	39,412	0.003952569
PC(p-38:3)/PC(o-38:4)A	20,181	64,890	0.004202996
PC(p-32:1)/PC(o-32:2)	9,363	20,474	0.004275232
PC 38:1	16,263	42,593	0.005217356
PC 37:6	11,239	35,212	0.005279249
PC(16:0_20:4)	28,931,051	53,402,365	0.005752917
PC 37:5	47,580	116,619	0.005997394
PC(p-38:5)/PC(o-38:6)	158,770	307,694	0.008146112
PC(18:2_18:2)	22,306,084	41,033,132	0.008743996
PC(16:0_22:6)	15,035,676	25,864,778	0.010461728
PC(p-34:1)/PC(o-34:2)	40,587	110,459	0.011318898
PC(p-42:5)/PC(o-42:6)	22,624	47,197	0.015107157
PC(16:0_18:1)	14,005,109	28,988,308	0.01571763
PC(18:2_20:4)	11,043,269	19,331,439	0.015779177
PC(18:3_18:3)	7,190	15,091	0.017188207
PC(p-34:2)/PC(o-34:3)	12,575	34,342	0.017422368
PC(14:0_18:2)	42,618	102,328	0.019365582
PC(p-38:4)/PC(o-38:5)	162,550	299,056	0.022117396
PC(p-34:0)/PC(o-34:1)	82,298	132,534	0.023567292
PC(p-36:4)/PC(o-36:5)	128,403	252,282	0.02378729
PC(20:0_18:2)	118,694	224,791	0.025843285
PC(16:0_22:4)	566,758	1,098,135	0.02940295
PC(16:1_18:2)	220,162	292,355	0.033872581
PC(p-38:6)/PC(o-38:7)	69,692	100,671	0.035179669
PC(p-36:1)/PC(o-36:2)	20,699	39,588	0.037242501
PC(18:2_18:3)A	87,828	38,090	0.039675013

## Validation of protocol

This protocol was used to generate Figure 3 and associated supplementary files within the following publication: Seufert et al. (2022).

## General notes and troubleshooting

Researchers may want to determine which anticoagulant will best fit their experimental design (e.g., heparin vs. EDTA), as some are known to influence lipid extraction and MS analysis (Gonzalez-Covarrubias et al., 2013). Importantly, the same anticoagulant and amount should be used throughout the study in order to avoid potential differences that may occur due to the use of anticoagulants in sample collection.

1. Alternative mouse models and diets can be used specific to the experimental question.
2. Newer versions of software may also be used.
3. Knowledge of MultiQuant software is required.
4. An in-depth knowledge of R and bioinformatics is required for the data analysis. All code and datasets for Figure 5 can be accessed at https://github.com/hickman6/Lipidomics_Code.
